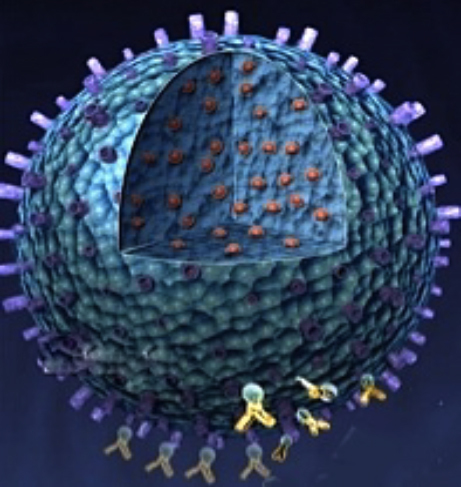# Therapeutic stromal nanoparticles for Parkinson’s disease

**Published:** 2014-10

Cell-replacement approaches represent a promising strategy to substitute degenerating dopaminergic (DA) neurons in Parkinson’s disease (PD). However, long-term survival of transplanted cells is still limited by the lack of both adequate growth factor supply and stromal support. To overcome this, Su Metcalfe and colleagues developed a surrogate nano-stroma using biodegradable nanoparticles, which were preloaded with leukaemia inhibitory factor (LIF), a proneural and reparative factor. When targeted to human fetal DA neurons, LIF-nano-stroma improved the survival of these cells both *ex vivo* and after *in vivo* transplantation. In addition, the authors used XAV939 – an inhibitor of the Wnt–β-catenin signalling pathway, which is involved in neural development – and found that XAV939-nano-stroma promoted the survival and differentiation of human fetal DA precursors. This study shows that stromal nanoparticles can be used to successfully deliver pro-survival cargos to single cells. Such an approach could improve the efficacy of cell-based transplantation therapies for human neurodegenerative diseases, including PD. **Page 1193**

**Figure:**